# Lung Ultrasound for Detection of Pulmonary Complications in Critically Ill Obstetric Patients in a Resource-Limited Setting

**DOI:** 10.4269/ajtmh.20-0996

**Published:** 2020-12-14

**Authors:** Luigi Pisani, Anna De Nicolo, Marcella Schiavone, Adetunji O. Adeniji, Angela De Palma, Francesco Di Gennaro, Edward Ejiro Emuveyan, Salvatore Grasso, Patricia C. Henwood, Alimamy P. Koroma, Stije Leopold, Claudia Marotta, Giuseppe Marulli, Giovanni Putoto, Enzo Pisani, James Russel, Ary Serpa Neto, Arjen M. Dondorp, Eva Hanciles, Michael M. Koroma, Marcus J. Schultz

**Affiliations:** 1 Department of Intensive Care, Amsterdam University Medical Centers–Location AMC, Amsterdam, The Netherlands;; 2 Mahidol–Oxford Tropical Medicine Research Unit (MORU), Mahidol University, Bangkok, Thailand;; 3 Intensive Care Unit, Department of Emergency and Organ Transplantation (DETO), University of Bari, Bari, Italy;; 4 Thoracic Surgery Unit, Department of Emergency and Organ Transplantation (DETO), University of Bari, Bari, Italy;; 5 Princess Christian Maternity Hospital, Freetown, Sierra Leone;; 6 Section of Operational Research, Doctors with Africa–Cuamm, Padova, Italy;; 7 Department of Emergency Medicine, Thomas Jefferson University Hospitals, Thomas Jefferson University, Philadelphia, Pennsylvania;; 8 Department of Internal Medicine, Amsterdam University Medical Centers – Location AMC, Amsterdam, The Netherlands;; 9 Department of Cardiology, Connaught Hospital, University of Sierra Leone, Freetown, Sierra Leone;; 10 Department of Critical Care Medicine, Hospital Israelita Albert Einstein, São Paulo, Brazil;; 11 Nuffield Department of Medicine, University of Oxford, Oxford, United Kingdom;; 12 Department of Anesthesia and Intensive Care, Connaught Hospital, University of Sierra Leone, Freetown, Sierra Leone;; 13 Laboratory of Experimental Intensive Care and Anesthesiology (L·E·I·C·A), Amsterdam University Medical Centers – Location AMC, Amsterdam, The Netherlands

## Abstract

Critically ill parturients have an increased risk of developing pulmonary complications. Lung ultrasound (LUS) could be effective in addressing the cause of respiratory distress in resource-limited settings with high maternal mortality. We aimed to determine the frequency, timing of appearance, and type of pulmonary complications in critically ill parturients in an obstetric unit in Sierra Leone. In this prospective observational study, LUS examinations were performed on admission, after 24 and 48 hours, and in case of respiratory deterioration. Primary endpoint was the proportion of parturients with one or more pulmonary complications, stratified for the presence of respiratory distress. Secondary endpoints included timing and types of complications, and their association with “poor outcome,” defined as a composite of transfer for escalation of care or death. Of 166 patients enrolled, 35 patients (21% [95% CI: 15–28]) had one or more pulmonary complications, the majority diagnosed on admission. Acute respiratory distress syndrome (period prevalence 4%) and hydrostatic pulmonary edema (4%) were only observed in patients with respiratory distress. Pneumonia (2%), atelectasis (10%), and pleural effusion (7%) were present, irrespective of respiratory distress. When ultrasound excluded pulmonary complications, respiratory distress was related to anemia or metabolic acidosis. Pulmonary complications were associated with an increased risk of poor outcome (odds ratio: 5.0; 95% CI: 1.7–14.6; *P* = 0.003). In critically ill parturients in a resource-limited obstetric unit, LUS contributed to address the cause of respiratory distress by identifying or excluding pulmonary complications. These were associated with a poor outcome.

## INTRODUCTION

Maternal mortality in Sierra Leone is among the highest in the world, with 1,360 maternal deaths/100,000 babies born alive.^1^ Major direct obstetric complications are an important cause of increased mortality.^2^ The three leading causes of mortality and morbidity during pregnancy are peripartum hemorrhage, sepsis, and preeclampsia^3,4^—each of these directly or indirectly predispose for pulmonary complications.^3^ Pulmonary complications requiring transitory or intense critical care treatment may arise before, during, and even after the primary obstetric disease is resolved. Among obstetric patients in Sierra Leone, additional risk factors for developing pulmonary complications include pregnancy-associated cardiopulmonary changes,^5^ iatrogenic fluid overload, tocolytic therapy,^5^ transfusion-related acute lung injury in patients who receive blood transfusions,^6^ and sickle cell disease.^7^

Bedside imaging techniques are increasingly available to detect pulmonary pathologies, including point-of-care lung ultrasound (LUS).^8,9^ Lung ultrasound is a low-cost, repeatable, and radiation-free imaging technique with a steep learning curve. It is an example of frugal innovation in critical care, thus sustainable also in resource-limited settings where conventional radiological tools are absent.^10^ Besides, X-ray imaging is preferably restricted in parturients because of the ionizing risk on the fetus. Lung ultrasound patterns are usually normal in parturients during the last gestational weeks,^11^ allowing the detection of acute pulmonary abnormalities. A recent hospital-wide study performed in Rwanda found that acute respiratory distress syndrome (ARDS) detected by LUS was a frequent and often lethal complication.^12^ Up to one in every 10 patients with ARDS in that study was obstetric, of whom 20% died.

The frequency with which ARDS and other pulmonary complications develop and are associated with poor outcome in critically ill parturients is largely unknown in resource-limited settings with a high maternal mortality. The objectives of the current study were to determine frequency, timing with regard to admission, type of pulmonary abnormalities detectable by LUS, and their associations with poor outcome in parturients admitted to the high dependence unit (HDU) of a large urban maternity hospital in Freetown, Sierra Leone. The primary hypothesis tested is that a large proportion of patients develop pulmonary complications identifiable by LUS and that development of these complications is associated with poor outcome.

## METHODS

### Design and ethical approval.

This was a prospective observational study conducted between July 2018 and February 2019 in critically ill parturients admitted to the HDU of the Princess Christian Maternity Hospital (PCMH) in Freetown, Sierra Leone. The study was approved by the Sierra Leone Ethical Research Committee on June 5, 2018. A waiver of written consent was granted because of the observational purpose of the study. The study was registered at clinicaltrials.gov (study identifier NCT 03828630).

### Patients.

Patients were eligible for participation if 1) parturient and 2) admitted to the HDU of the PCMH. Patients were excluded if they had passed the time window of 6 hours after admission to the HDU. Anticipated logistical reasons for exclusion were the nonavailability of the physician sonographer, for example, during weekend days, or when the dedicated physician was on call in the operating room or another ward. Patients were stratified by the presence of respiratory distress at any point during HDU stay.

### Data collected.

Patient clinical and ultrasound granular data were collected at predefined timepoints: on admission, after 24 hours and 48 hours, and at any point in case of patient deterioration. Demographic data collected on admission included age, weight, height, reported reason for hospital and for HDU admission, and the women gravidity, parity, and gestational age; malaria status and preexistent comorbidities; and surgical and transfusion status during the current hospital admission.

Vital signs, including heart rate, respiratory rate, temperature, neurological status, and systolic and diastolic arterial blood pressure, were captured at the moment of each LUS examination and used to compute the modified obstetric early warning score (OEWS).^13^ Obstetric early warning score is a composite score reflecting impairment of physiological parameters at admission. Also, peripheral pulse oximetry oxygen saturation (SpO_2_), findings of chest auscultation, use of accessory muscles, and presence of nasal flaring were collected at each time point. Whenever performed for clinical reasons, point-of -care laboratory measures such as hemoglobin, glucose, and capillary lactate levels were recorded. However, no systematic laboratory examination was performed for the study purpose. Urinary output was recorded at each timepoint for catheterized patients together with the amounts of intravenous fluids administered at 24 and 48 hours. It was also recorded whether supplementary oxygen, vasopressors, and diuretics were administered. Follow-up ended at HDU discharge. Then, it was recorded whether the patients left the HDU alive or not, and status at discharge, that is, improved and discharged to ward, or transfer to tertiary hospital because of escalation of care.

### Primary and secondary endpoints.

The primary endpoint was the proportion of parturients with pulmonary complications detectable by LUS during stay in HDU. Secondary endpoints were the timing of appearance and types of pulmonary complications, the patients’ global LUS aeration score, and occurrence of poor outcome.

### The protocol for LUS.

**Figure 1.**
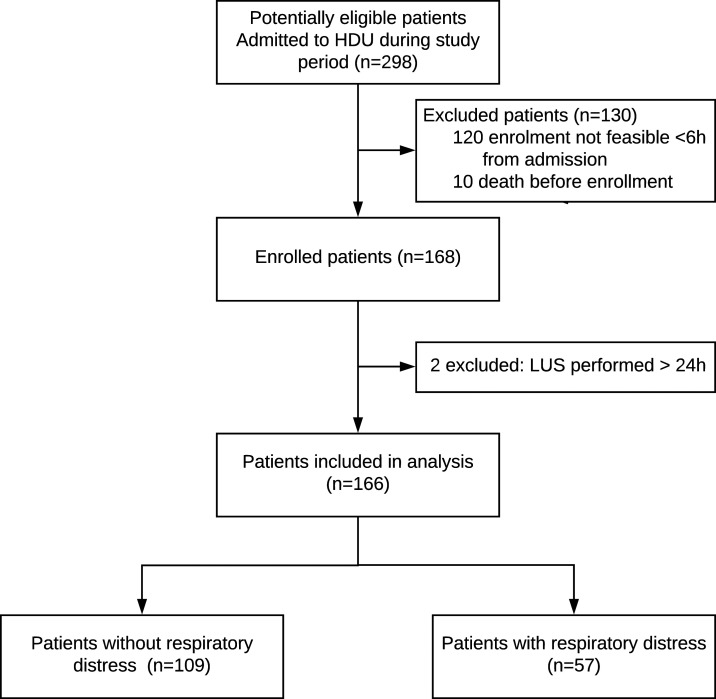
Patient flowchart. HDU = high dependency unit; LUS = lung ultrasound.

A comprehensive LUS examination was performed at each timepoint and in case of respiratory distress or clinical pulmonary edema during HDU stay (Figure 1). To minimize operator dependency and bias, LUS was performed using standardized operating procedures and structured region-based case report forms. All LUS examinations were performed by senior residents in intensive care medicine or thoracic surgery (A. d. N., M. S.), with a > 2-year experience in ultrasound procedures in critically ill patients. Dedicated bedside and remote training on at least 12 supervised examinations^14^ was performed together with an experienced LUS sonographer (L. P.) to familiarize with the systematic LUS scoring and case report form completion. Lung ultrasound was performed using a MyLab^™^Five ultrasound machine (Esaote Spa, Genova, Italy) and a low frequency (2.5–5 MHz) convex probe. The ultrasound machine, the probe, and the probe holder were disinfected before and after each use as from hospital indications. The patient remained in supine or semi-recumbent position, and the probe was held perpendicular to the skin.

The 12-region technique was used, in which ultrasound was performed on six areas on each side of the chest, that is, two ventral regions, two lateral regions, and two posterolateral regions, with a transversal approach to maximize lung exposition and minimize rib-related artifacts.^15^ The examiner scored the worst aeration pattern observed in each region using the LUS aeration score.^16^ Each lung field was scored from 0 to 3 as follows: “0,” A-pattern with ≤ 2 B-lines; “1,” more than 2 separated B-lines; “2,” multiple coalescent B-lines; or “3,” lung consolidation, defined as anechoic or tissue-like images arising from the pleural line that is limited in depth by an irregular border. A global LUS score was calculated at each time point and ranged from 0 to 36. The presence of subpleural consolidations, effusion, air bronchograms, and abnormal pleural line was assessed in each field. Normal lung aeration observed using LUS was defined as all lung fields normally aerated, that is, with an A-pattern or aeration score of 0; bilateral interstitial syndrome was defined as the presence of two or more regions with a B-pattern (score 1 or 2) per hemithorax.^8,17^ A subcostal view was also acquired with the maximal and minimal diameter of the inferior vena cava, measured in motion mode 2 cm distal to the origin of the right atrium.^18^ Twelve ultrasound clips of 5 seconds each were stored after each examination on the ultrasound machine and exported as video files to a portable computer for offline analysis and quality control purposes.

### Definitions.

Respiratory distress was defined as the presence of one or more among the following criteria: respiratory rate ≥ 30 breaths/minute, SpO_2_/fraction of inspired oxygen (FiO_2_) ≤ 315, and signs of difficult breathing, including the use of accessory muscles or nasal flaring.

The following definitions were used to derive the pulmonary complications from the collected clinical and ultrasound data.

Acute respiratory distress syndrome––defined according to the Kigali modification of the Berlin definition for ARDS.^12^ The oxygenation status was computed as the ratio between SpO_2_ and FiO_2_. Fraction of inspired oxygen was derived from the oxygen flow using the formula FiO_2_ = 0.21 + (O_2_ flow × 0.03).^12^ Bilateral opacities on LUS were defined as the presence of interstitial syndrome and/or two or more regions with lung consolidation bilaterally, not fully explained by effusions, lung collapse, or nodules. To fulfill the origin of edema criteria (i.e., respiratory failure not fully explained by cardiac failure or fluid overload) in a setting with absent echocardiography, all patients with a known history of cardiac failure or diagnosed with fluid overload were excluded from this potential diagnosis.

Fluid overload or hydrostatic pulmonary edema––defined as the presence of a bilateral interstitial syndrome or pleural effusion on LUS, associated with a positive fluid balance (> 1,000 mL in the last 24 hours), and/or a maximal diameter of the inferior vena cava of > 23 mm.^19^

Pneumonia––defined as focal or multifocal interstitial syndrome and/or consolidation on LUS plus at least one of the following: temperature > 38.3°C or white blood cell count > 12,000/mm^3^ (if available).

Atelectasis––defined as the presence of mono or bilateral focal consolidations, denoting a focal loss of aeration, which did not fall in the case definitions for ARDS, pneumonia, or fluid overload.

Pleural effusion––defined as hypoechoic or anechoic collection between the parietal and visceral pleura in at least one lung region.^17^

Poor outcome––a composite of transfer for escalation of care or death in the HDU.

### Power calculation.

All patients admitted over the time span the study ran were to be included. Considering previous semester admission rates and operator availability, it was expected that more than 150 patients would be eligible during the predefined 6 months recruitment period. Considering an estimated prevalence of 20%, this sample size allows to estimate the proportion of pulmonary complications with 5% precision and 95% CI in a finite population.^20^

### Statistical analysis.

Demographic, clinical, and outcome variables were presented as percentages for categorical variables and as medians with interquartile ranges for continuous variables.

Patients were stratified according to the presence or absence of respiratory distress. The proportion of patients with pulmonary complications during the HDU stay was calculated as the number of patients suffering from at least one pulmonary complication divided by the total number of patients enrolled in the study, and by the number of patients in the group with and without respiratory distress. Types of pulmonary complications and individual frequencies were reported separately for the group with and without respiratory symptoms. The chi-square statistics was used to seek significant differences across patient groups for categorical endpoints.

The Mann Whitney *U* test and the Kruskal–Wallis test were used to compare LUS scores and other numerical variables between patients with and without respiratory symptoms on admission. Interobserver variability for the LUS scoring between the study sonographers and the expert scorer was assessed on 320 LUS images and expressed as Fleiss’ kappa statistics.

A logistic regression model was used to test potential associations between occurrence of pulmonary complications and a poor outcome, defined as death in the HDU or transfer for escalation of care. The model was corrected for severity of illness on admission as estimated by the OEWS.

All statistical analyses were performed in *R* (version 3.3.1, www.r-project.org, R Core Team, Vienna, Austria) and graphs built using GraphPad Prism (version 7.03, www.graphpad.com, GraphPad Software, San Diego, CA). A *P*-value below 0.05 was considered significant.

## RESULTS

### Patient cohort.

**Figure 2.**
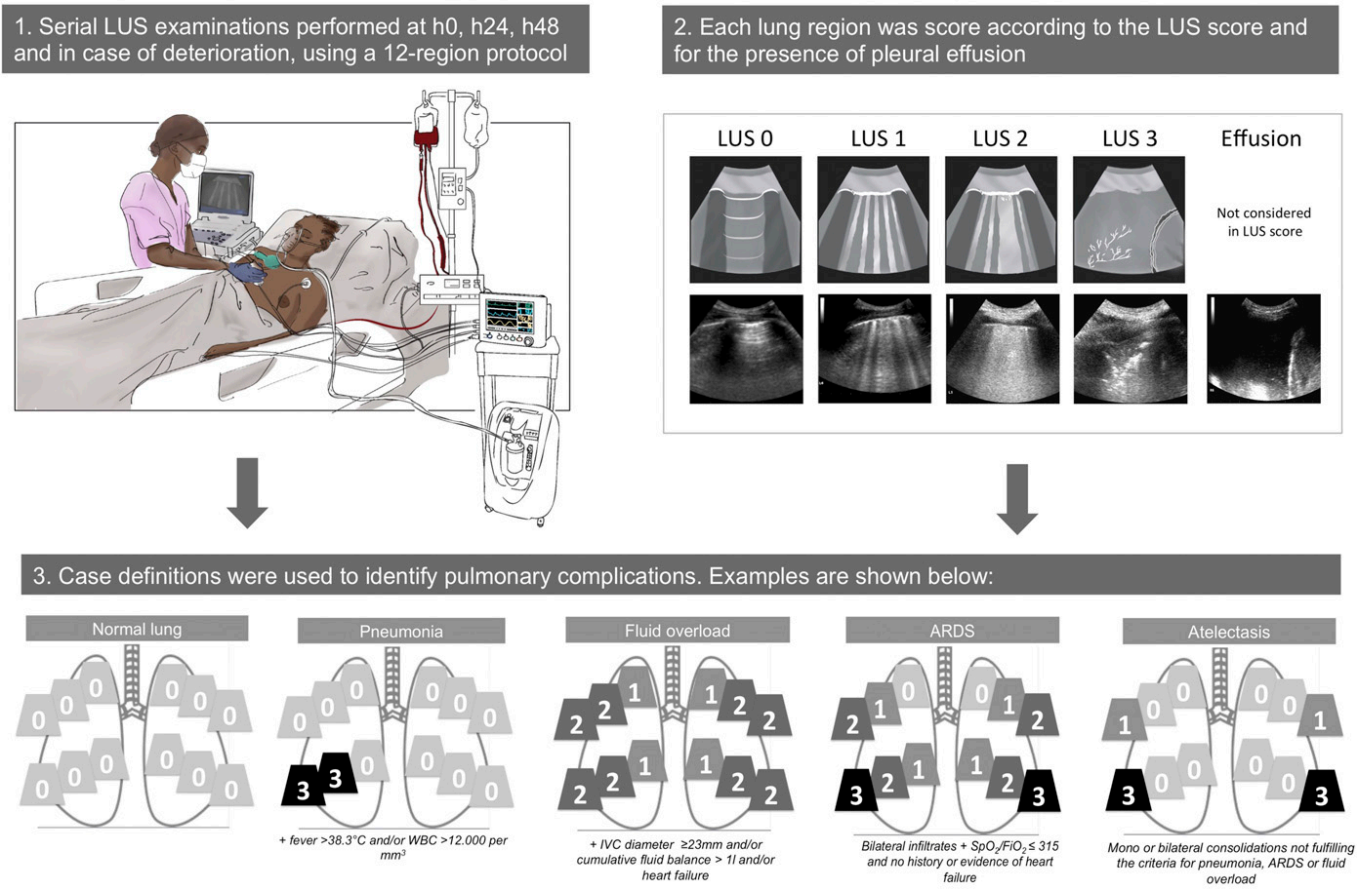
Lung ultrasound protocol including the LUS score assessment and predefined case definitions of pulmonary complications integrating LUS and clinical data. One example for each pulmonary complication case definition is shown. Lung ultrasound images were drawn from the study saved clips, with additional details in the text. FiO_2_ = fraction of inspired oxygen; IVC = inferior vena cava; LUS = lung ultrasound; SpO_2_ = peripheral pulse oximetry oxygen saturation. This figure appears in color at www.ajtmh.org.

Of 298 potentially eligible patients, 166 patients were enrolled in the study. Patient flow is detailed in Figure 2. Baseline characteristics are reported in Table 1. Of all patients, 34% presented with or developed respiratory distress during HDU stay. In total, 18 of 166 patients met the composite endpoint of a poor outcome (11%); transfer for escalation of care occurred in 10 (6%), whereas mortality was eight (5%).

**Table 1:** Patient characteristics at baseline

Variable	Number
General epidemiology	
Age (years)	25 (22, 30)
Gestation age (weeks)	36 (32, 38)
Gravidity	3 (2,4)
Parity	1 (0,3)
Body mass index (kg/m^2^)	23.9 (22.2, 25.7)
Clinical features	
Obstetric early warning score total score	3 (2, 4)
Altered sensorium, *n* (%)*	16 (9.6)
Mean arterial blood pressure (mmHg)†	95 (82, 110)
Heart rate (beats/minute)‡	105 (95, 120)
Respiratory features	
Respiratory rate (movements/minute)	24 (22, 28)
SpO_2_	99 (98, 99)
SpO_2_/fraction of inspired oxygen	467 (462, 471)
Oxygen therapy, yes, *n* (%)	29 (17.4)
Biology	
Hemoglobin (g/dL)§	7.9 (6.3, 9.8)
Capillary lactates levels (mmol/L)‖	4.5 (2.6, 7.1)
Positive to malaria, *n* (%)	12 (7.2)
Reason of admission, *n* (%)	
Antepartum hemorrhage	45 (28.7)
Postpartum hemorrhage	14 (8.9)
Uterine rupture	22 (14)
Severe preeclampsia	30 (19.1)
Obstructed labor	15 (9.6)
Ectopic pregnancy	7 (4.5)
Sepsis	11 (7.0)
Sickle cell disease	5 (3.2)
Type of delivery/surgery, *n* (%)	
Spontaneous vaginal delivery	17 (10.8)
Cesarean section	68 (43.3)
Surgery (other than cesarean)	37 (23.6)
Type of anesthesia, *n* (%)	
General anesthesia	84 (53.5)
Spinal anesthesia	18 (11.5)

SpO_2_ = peripheral pulse oximetry oxygen saturation. Data are presented as median (interquartile range) or proportion (%).

* Patients only responsive to painful stimulus or unresponsive on admission.

† Missing in three patients.

‡ Missing in one patients.

§ Missing in five patients.

‖ Missing in 46 patients.

### Lung ultrasound examinations and interobserver agreement.

A total of 383 LUS examinations, median two (2–3) LUS/patient, were performed. All 166 patients were scanned at the day of admission, 121 also after 24 hours, and 86 also after 48 hours. Ten additional LUS examinations were performed in patients who deteriorated in between these planned examinations. Of all potential lung regions, 39 (< 1%) regions could not be examined because of surgical dressings or patient position. Agreement between the two study sonographers and the expert scorer in recording the LUS score for individual lung fields was kappa 0.77. Kappa statistics for anterior, lateral, and posterior lung zones were 0.76, 0.75, and 0.78, respectively.

### Prevalence and timing of pulmonary complications.

Overall, 21% (95% CI: 15–28) of patients had at least one pulmonary complication detectable by LUS during HDU stay. The incidence of pulmonary complications was higher in patients with respiratory distress versus in patients not having respiratory distress (21 of 57 [37%] versus 14 of 109 [13%]; *P* = 0.001).

Timing of occurrence of pulmonary complications was similar between patients with respiratory distress (67% on admission, and 24% and 10% in the first 24 hours or thereafter) and patients not having respiratory distress (57% on admission, 36% and 7% in first 24 hours or thereafter).

### Types of pulmonary complications and patient characteristics.

Types and frequency of pulmonary complications are detailed in Table 2. Clinical characteristics are reported in Table 3. Patients with respiratory distress who had pulmonary complications detected by LUS were commonly hypoxemic and more often transferred for escalation of care. Patients with respiratory distress in the absence of pulmonary complication frequently had hemodynamic compromise in terms of higher heart rate and capillary lactates. The few patients who had a complication in the absence of respiratory distress usually had higher body mass index (BMI) and suffered from atelectasis. Most patients never developed respiratory distress and had negative LUS examinations.

**Table 2:** Types of pulmonary complications detected by LUS in patients with and without respiratory distress, and associated patients’ outcomes

	Respiratory distress (*n* = 57)		No Respiratory distress (*n* = 109)		*P*-value
	With LUS complication (*n* = 21)	No LUS complication (*n* = 36)	With LUS complication (*n* = 14)	No LUS complication (*n* = 95)	
Pulmonary complications,* *n* (%)					< 0.001
Acute respiratory distress syndrome	6 (28.6)	–	0 (0)	–	
Fluid overload	6 (28.6)	–	0 (0)	–	
Effusion	8 (38.1)	–	3 (21.4)	–	
Pneumonia	2 (9.5)	–	1 (7.1)	–	
Atelectasis	6 (28.6)	–	11 (78.6)	–	
Outcomes					
Length of stay (days)	3.0 (2.0,3.2)	3.0 (2.0, 4.2)	3.5 (3.0, 4.0)	3.0 (2.0, 4.0)	0.541
Poor outcome, *n* (%)	6 (28.6)	5 (13.9)	3 (21.4)	4 (4.2)	0.004
Death, *n* (%)	0 (0)	5 (13.9)	1 (7.1)	2 (2.1)	–
Transfer, *n* (%)	6 (28.6)	0 (0)	2 (14.3)	2 (2.1)	–

LUS = lung ultrasound. Data are presented as median (interquartile range) or proportion (%).

* Nonexclusive categories.

**Table 3:** Patient characteristics in the different study groups

	Respiratory distress (*n* = 57)		No Respiratory distress (*n* = 109)		*P*-value
	With LUS complication (*n* = 21)	No LUS complication (*n* = 36)	With LUS complication (*n* = 14)	No LUS complication (*n* = 95)	
Clinical features					
Obstetric early warning score total score	3 (2, 5)	4 (2, 6)	2 (2, 3)	3 (2, 4)	0.003
Body mass index (kg/m^2^)	23.4 (22, 25)	24.2 (23.3, 25.9)	25.1 (23.4, 28.8)	23.4 (21.5, 25.3)	0.03
Respiratory rate (movements/minute)	28 (24, 34)	31 (24, 36)	23 (22, 25.5)	24 (22, 25)	< 0.001
Oxygen therapy, yes, *n* (%)	14 (66.7)	15 (41.7)	0 (0)	0 (0)	< 0.001
Peripheral pulse oximetry oxygen saturation/fraction of inspired oxygen	275 (269, 452)	462 (274, 471)	467 (467, 470)	471 (467, 471)	< 0.001
Altered sensorium, *n* (%)*	5 (23.8)	14 (40.0)	3 (21.4)	18 (19.4)	0.114
MAP (mmHg)	85 (81, 112)	95 (78, 105)	89 (82, 108)	95 (83, 109)	0.87
Heart rate (beats/minute)	105 (90, 125)	115 (103, 130)	98 (81, 114)	102 (95, 113)	0.012
Biology, *n* (%)					
Hemoglobin (g/dL)	9.4 (7, 10.4)	7.1 (5.7, 10.0)	7.6 (5.9, 8.9)	8.0 (6.4, 9.6)	0.466
Cap. lactates, (mmol/L)†	3.5 (2.5, 5.0)	4.8 (3.2, 8.2)	2 (1.9, 3.7)	4.8 (2.9, 7.3)	0.023
Positive to malaria	5 (23.8)	4 (11.1)	2 (14.3)	2 (2.1)	0.004
Reason of admission, *n* (%)					
Antepartum hemorrhage	14 (66.7)	16 (44.4)	3 (21.4)	14 (14.7)	< 0.001
Postpartum hemorrhage	1 (4.8)	1 (2.8)	1 (7.1)	12 (12.6)	0.291
Uterine rupture	1 (4.8)	4 (11.1)	0 (0)	18 (18.9)	0.112
Severe preeclampsia	5 (23.8)	6 (16.7)	5 (35.7)	15 (15.8)	0.301
Obstructed labor	2 (9.5)	4 (11.1)	0 (0)	11 (11.6)	0.61
Ectopic pregnancy	0 (0)	2 (5.6)	0 (0)	5 (5.3)	0.581
Sepsis	1 (4.8)	4 (11.1)	2 (14.3)	4 (4.2)	0.319
Sickle cell disease	1 (4.8)	4 (11.1)	0 (0)	0 (0)	0.009
Procedures, *n* (%)					
Type of delivery					0.155
Spontaneous vaginal delivery	3 (14.3)	4 (11.1)	1 (7.1)	10 (10.5)	
Cesarean section	6 (28.6)	9 (25)	6 (42.9)	51 (53.7)	
Surgery (other than cesarean section)	1 (4.8)	7 (19.4)	1 (7.1)	30 (31.6)	0.019
General anesthesia	3 (14.3)	13 (36.1)	8 (57.1)	66 (69.5)	< 0.001
Management features first 24 hours, *n* (%)					
Transfusion	8 (38.1)	12 (33.3)	9 (64.3)	51 (53.7)	0.084
Fluids administered (mL)‡	2,500 (1725, 3,775)	2,100 (1,500, 2,700)	1,700 (1,250, 2,450)	1,975 (1,413, 2,450)	0.266
Use of vasopressors	3 (14.3)	4 (11.1)	0 (0)	1 (1.1)	0.013

LUS = lung ultrasound; MAP = mean arterial pressure. Data are presented as median (interquartile range) or proportion (%).

* Patients only responsive to painful stimulus or unresponsive on admission.

† Measurement available in 120 patients.

‡ Assessed in 126 patients.

### Lung ultrasound score.

**Figure 3.**
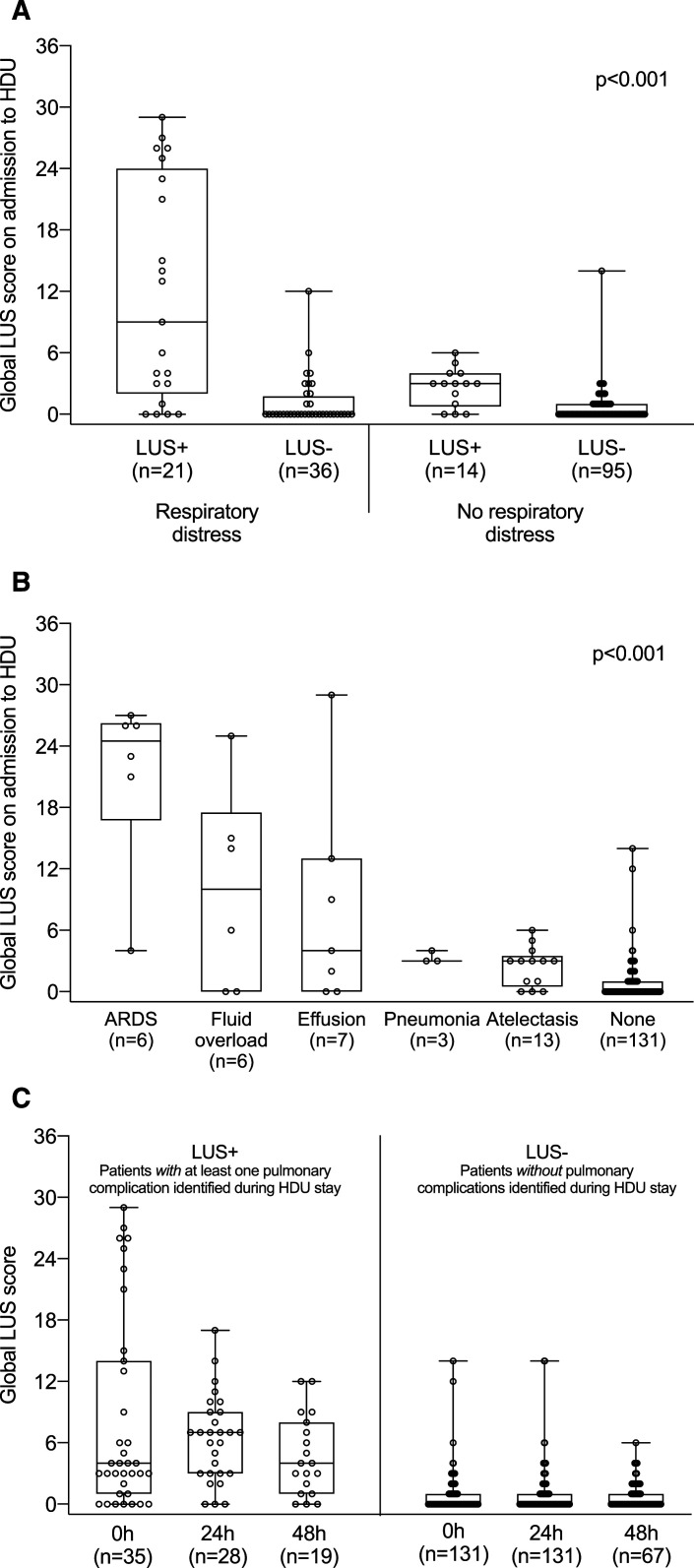
Baseline global lung ultrasound score on patient admission expressing loss of lung aeration in the different patient groups (**A**) and across different pulmonary complications (**B**). Individual global lung ultrasound scores are also represented at admission, at 24 hours and 48 hours (**C**). The middle line represents the median, the lower hinge represents the first quartile, the upper hinge represents the third quartile, and the whiskers extend to the minimum and maximum values. ARDS = acute respiratory distress syndrome; LUS = lung ultrasound.

Overall, LUS examinations in which a pulmonary complication was detected had higher global LUS score than negative LUS examinations (four [3–9] versus zero [0–1]; *P* < 0.001). The baseline global LUS score was different in the four patient groups, and for different pulmonary complications (Figure 3).

### Association with outcome.

The occurrence of a poor outcome was highest in patients with respiratory distress and pulmonary complications detected by LUS (Table 2). The occurrence of one or more pulmonary complication was associated with poor outcome (odds ratio: 5.0; 95% CI: 1.7–14.6; *P* = 0.003).

## DISCUSSION

In this study of critically ill obstetric patients in a resource-limited HDU in Africa, pulmonary complications detectable by LUS were frequent, affecting one of five patients. Most of these pulmonary complications had an early onset. Acute respiratory distress syndrome and fluid overload occurred with equal prevalence. Presence or development of pulmonary complications diagnosed by LUS was associated with increased risk of poorer outcome.

Strengths of this study include the use of clear case definitions integrating clinical parameters with LUS variables. The prospective design and serial LUS follow-up allowed capturing complications that were not present on admission. Examinations were performed using an abdominal ultrasound probe that is widely available in obstetric units. The stratification by respiratory distress allowed investigation of the clinical meaning of LUS findings in critically ill parturients in a setting where resources are extremely limited.

Patients with abnormal lung findings identified through LUS and respiratory distress frequently had ARDS. The period prevalence of ARDS mirrors previous findings in a hospital-wide study in Rwanda.^12^ Whereas the absence of blood gas analysis and chest radiography did not allow to verify the diagnosis of ARDS against the current Berlin definition for ARDS,^21^ the used criteria were individually validated.^12,22^ Patients were exposed to ARDS risk factors such as sepsis, surgery, malaria, and whole blood transfusions,^23,24^ whereas pneumonia was rare.

Pulmonary edema was another diagnosis in patients with pulmonary complications and respiratory symptoms. The incidence of pulmonary edema in the current cohort is in line with estimates ranging from 0.1% in normal pregnancy to 10% in patients with preeclampsia.^25^ Physiologic changes of pregnancy, preeclampsia, and puerperal cardiomyopathy predispose parturients to pulmonary oedema.^5^ The combination of nifedipine use and magnesium sulfate, acute kidney injury related to severe preeclampsia and iatrogenic fluid overload may have contributed to pulmonary congestion.^26^

Lung ultrasound excluded a pulmonary complication in more than half of patients with respiratory distress. Lung ultrasound high negative predictive value is supported by findings in previous studies and allows to swiftly exclude parenchymal involvement in the dyspneic patient.^8,27^ These patients likely had other reasons to develop respiratory symptoms, such as metabolic acidosis and hemorrhagic anemia. Malaria may also induce compensatory tachypnea and represents a risk factor for lung injury.^28^ The exclusion of a pulmonary complication in dyspneic parturients was a key finding of this study and may provide the most added value in daily clinical practice in the obstetric population, which generally has a low pretest probability of pulmonary findings. Respiratory distress in combination with a negative LUS may also suggest a pulmonary embolism, which could have been captured with compression ultrasonography.^29^ However, to keep burden of the study acceptable, compression ultrasonography was not part of the study protocol.

Patients with a positive LUS in the absence of respiratory symptoms did not require oxygen and had normal oxygenation. Previous studies documented how diffuse B-lines may be present in asymptomatic parturients.^11,30^ The finding of frequent atelectasis in this group may be explained by the exposure to risk factors such as higher BMI and general anesthesia.^27,31^

Pulmonary complications were significantly associated with poorer outcome, confirming findings in non–obstetric patients in Africa^12^ and Asia.^32^ This does not imply a causal relationship with mortality, as parturients largely die of direct obstetric complications such as hemorrhage, eclampsia, and sepsis.^3,33^ Yet, pulmonary complications led to frequent escalation of care. Although most obstetric critical illness can be treated in medium care units,^3,34^ intensive care unit beds and mechanical ventilators are scarcely available in Africa.^3,35^ Identifying patients with complications may help the caregivers to allocate the scarce monitoring, oxygen, and ventilation resources.

This study faces limitations. A considerable number of patients were missed as died before recruitment or when it was not possible to perform LUS within 6 hours from admission because of logistical reasons. However, this strict predefined inclusion window was designed to pinpoint pulmonary conditions observed early in the course of obstetric critical illness. Pulmonary case definitions were defined from granular data at study completion. Although this minimizes observation bias, it may lead to under or overestimation of complications. No other radiological imaging techniques were available in this setting to confirm the LUS findings. This limitation is mitigated by the body of literature validating LUS against reference methods for the conditions investigated.^8,31,36^ Echocardiography was not available to definitively exclude a peripartum cardiomyopathy; thus, patients with a history of cardiac failure were excluded a priori from the diagnosis of ARDS. Similarly, compression ultrasonography for the exclusion of deep vein thrombosis was not performed. Finally, the analysis regarding impact of pulmonary complications was limited by the unknown survival status of transferred patients.

## CONCLUSION

In this cohort of critically ill obstetric patients in a resource-limited HDU, LUS was a useful and safe imaging tool to identify or exclude pulmonary involvement in patients with or without respiratory distress. Pulmonary complications occurred early, and their presence or development was associated with poor outcome.
